# There’s more than one way to climb a tree: Limb length and microhabitat use in lizards with toe pads

**DOI:** 10.1371/journal.pone.0184641

**Published:** 2017-09-27

**Authors:** Travis J. Hagey, Scott Harte, Mathew Vickers, Luke J. Harmon, Lin Schwarzkopf

**Affiliations:** 1 BEACON Center for Evolution in Action, Michigan State University, East Lansing, Michigan, United States of America; 2 School of Marine and Tropical Biology, James Cook University, Townsville, Queensland, Australia; 3 Centre for Tropical Biology and Climate Change, Commonwealth Scientific and Industrial Research Organization, Townsville, Queensland, Australia; 4 Department of Biological Sciences, University of Idaho, Moscow, Idaho, United States of America; Brown University, UNITED STATES

## Abstract

Ecomorphology links microhabitat and morphology. By comparing ecomorphological associations across clades, we can investigate the extent to which evolution can produce similar solutions in response to similar challenges. While *Anolis* lizards represent a well-studied example of repeated convergent evolution, very few studies have investigated the ecomorphology of geckos. Similar to anoles, gekkonid lizards have independently evolved adhesive toe pads and many species are scansorial. We quantified gecko and anole limb length and microhabitat use, finding that geckos tend to have shorter limbs than anoles. Combining these measurements with microhabitat observations of geckos in Queensland, Australia, we observed geckos using similar microhabitats as reported for anoles, but geckos with relatively longer limbs were using narrower perches, differing from patterns observed in anoles and other lizards. We also observed arboreal geckos with relatively shorter proximal limb segments as compared to rock-dwelling and terrestrial geckos, similar to patterns observed for other lizards. We conclude that although both geckos and anoles have adhesive pads and use similar microhabitats, their locomotor systems likely complement their adhesive pads in unique ways and result in different ecomorphological patterns, reinforcing the idea that species with convergent morphologies still have idiosyncratic characteristics due to their own separate evolutionary histories.

## Introduction

Ecomorphology is the study of morphology and performance in the context of ecology. Ecomorphological studies typically rely on correlations between morphology, performance, and habitat use to suggest adaptation [1–7], with lizards having been a classic system. Overall, researchers have described a wide range of patterns linking lizard locomotor morphology, performance, and microhabitat [8]. However, ecomorphological studies are typically limited to a clade of closely related species and general comparisons across distantly related groups are uncommon (but see [3]).

We investigated the extent of ecomorphological similarities between two distantly related groups of lizards, geckos and anoles. Anoles represent a well-studied example of ecomorphology, with correlated morphologies and ecologies having evolved repeatedly in Caribbean anoles. For example, anoles have repeatedly evolved shorter limbs in association with narrow perches. This correlation between relatively short limbs and narrow perches has also been observed for *Tropidurus* and *Draco* [9, 10], and is likely due to an interaction between sprint speed, balance, and limb length with perch diameter [6, 11–18]. Similar trade-offs between sprint speed and clinging ability have also been observed in chameleons [19, 20], suggesting that relatively short limbs may be a common adaptation associated with movement on narrow perches. While this ecological-morphological correlation has been observed across many groups of lizards, the repeatedly evolved Caribbean anole ecomorphs have not. Even closely related mainland anole species do not show the same ecomorphological patterns [21]. Alternatively, other studies have reported examples of distantly related ecomorphological convergence [3].

Given the ecological and morphological similarities between gecko and anole lizards, we were interested in investigating similarities in their ecomorphological traits, focusing on the relationship between limb length and microhabitat use. Geckos provide an excellent opportunity for comparison to anoles. Both geckos and anoles also exhibit fibrillar adhesive toe pads [22–29]. Although many studies have focused on the biomechanical properties of fibrillar toe pad adhesion [30–36], few have considered it in an ecological context [37–40] especially in the case of geckos (but see [25, 41–45]). Anoles are nearly all arboreal. Similarly, most pad bearing geckos are scansorial (climbing) using arboreal or saxicolous (rock dwelling) microhabitats [45–49]. Furthermore, similarities in habitat use patterns have previously been suggested between geckos and anoles [50, 51]. We hypothesized similar positive correlations between gecko limb length and arboreal perch diameter in light of the biomechanical trade-off between sprint speed and balance observed in anoles and other lizards [6, 9–20, 48, 52, 53].

## Materials and methods

**Fig 1:**
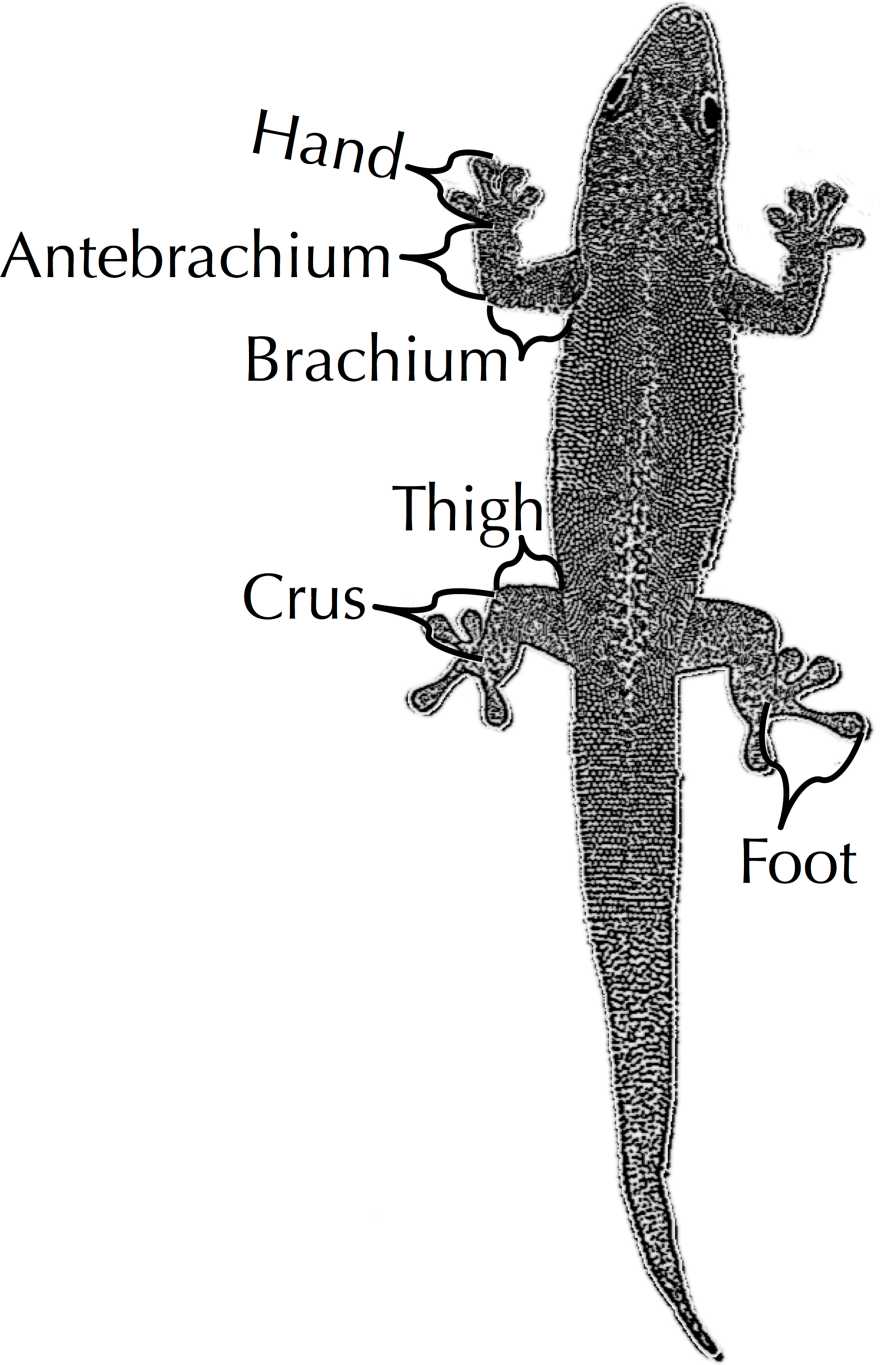
Limb measurements. Our limb measurements included hand length (from the center of the wrist joint to the tip of longest digit measured on the dorsal side), antebrachium length (from apex of the elbow joint to center of the wrist joint, on the dorsal side), brachium length (from the axilla to apex of the elbow joint), thigh length (from the point in which the hind limb enters the body to the apex of the knee); crus length (from the apex of the knee to the ankle joint); and foot length (from the center of ankle joint to the tip of longest digit, toe four, measured on the dorsal side).

**Fig 2:**
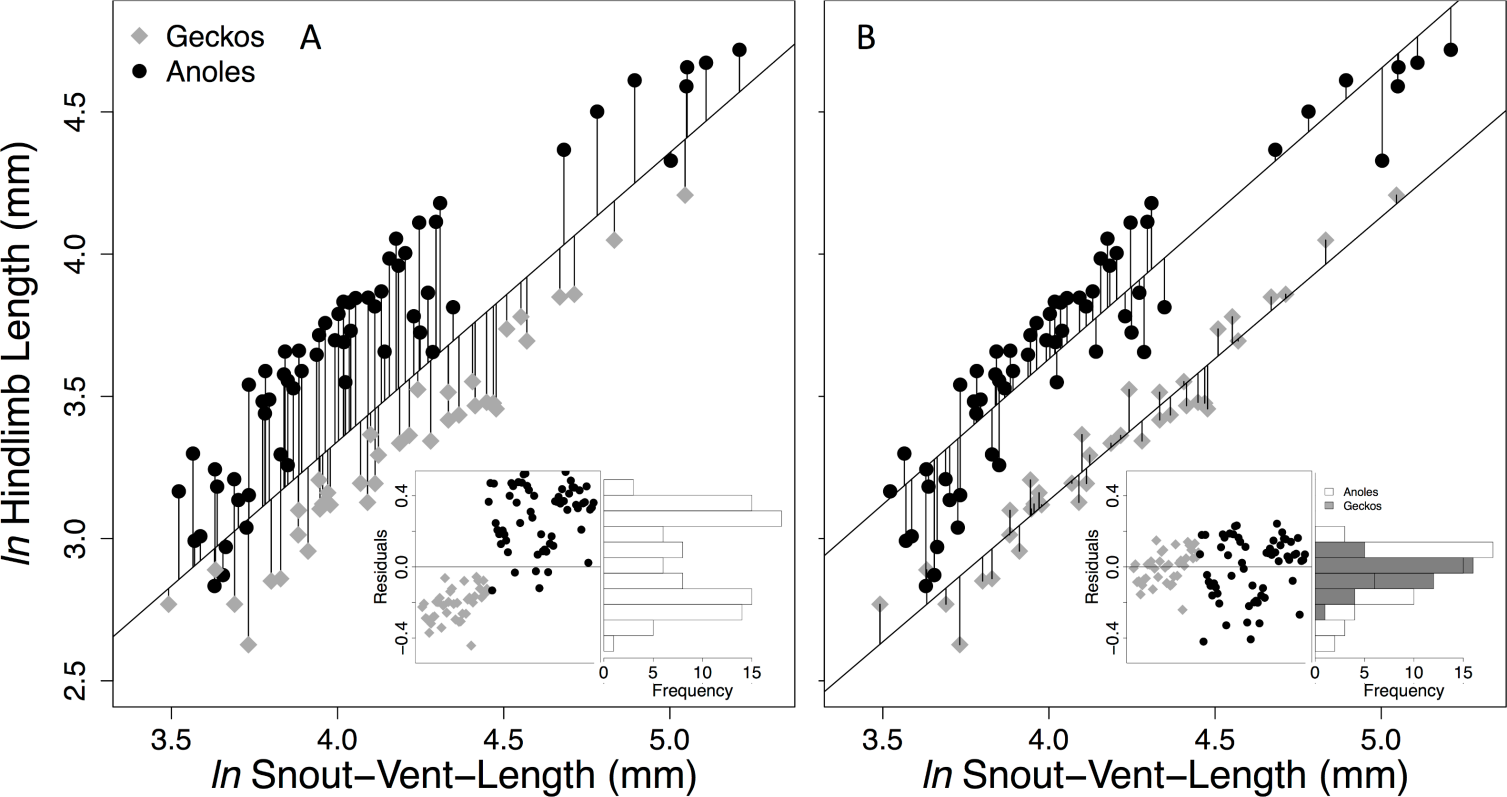
Body and total hind limb lengths. Pad-bearing gecko (grey) and anole (black) residuals from a single regression (A) and residuals from clade-specific regressions (B). Variation in residuals is shown in inserted scatter plots and horizontal bar graphs.

**Fig 3:**
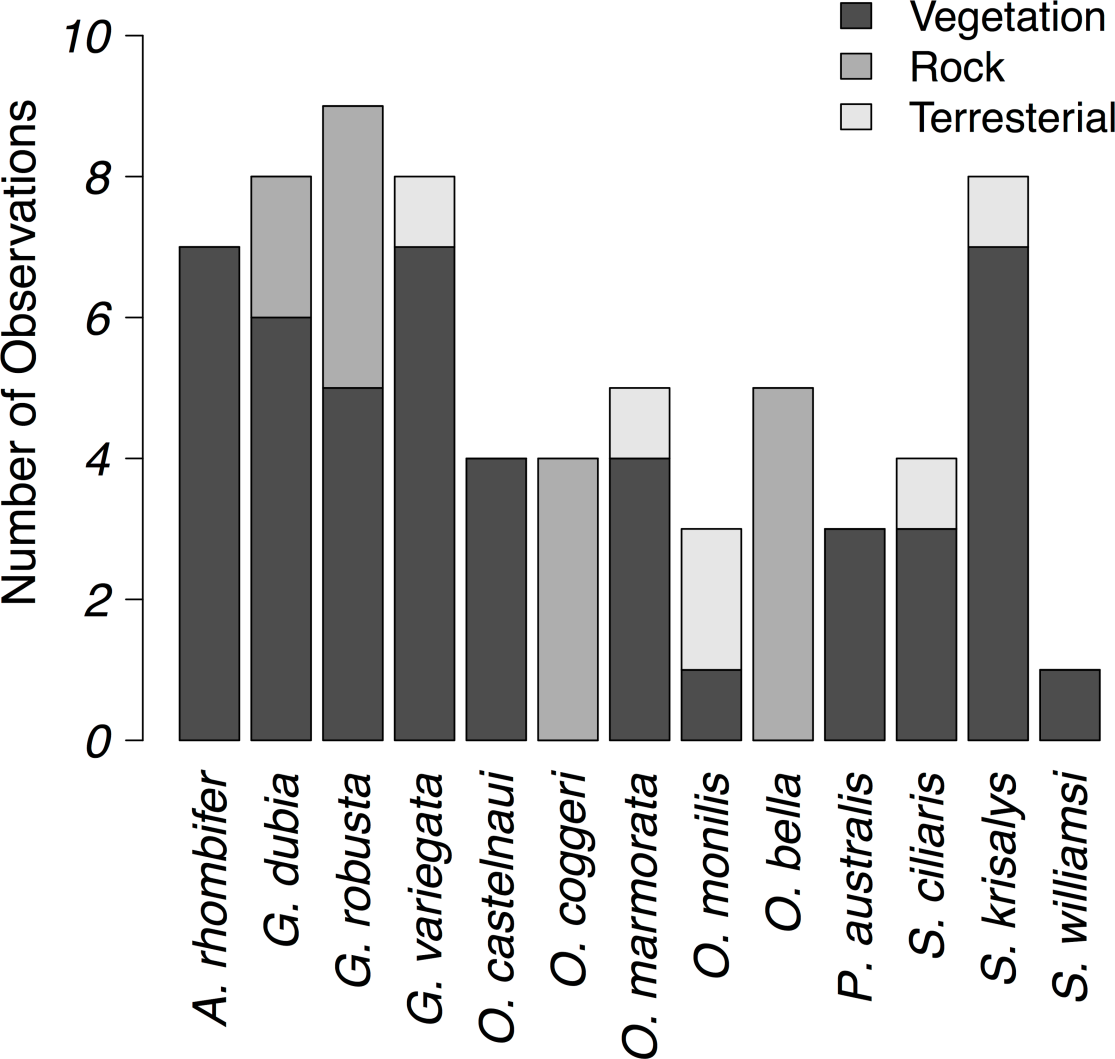
Perche types used by geckos observed in Queensland, Australia. The number of individual geckos observed using vegetation (dark gray), rocks (medium gray), and the ground (light gray) are shown.

**Fig 4:**
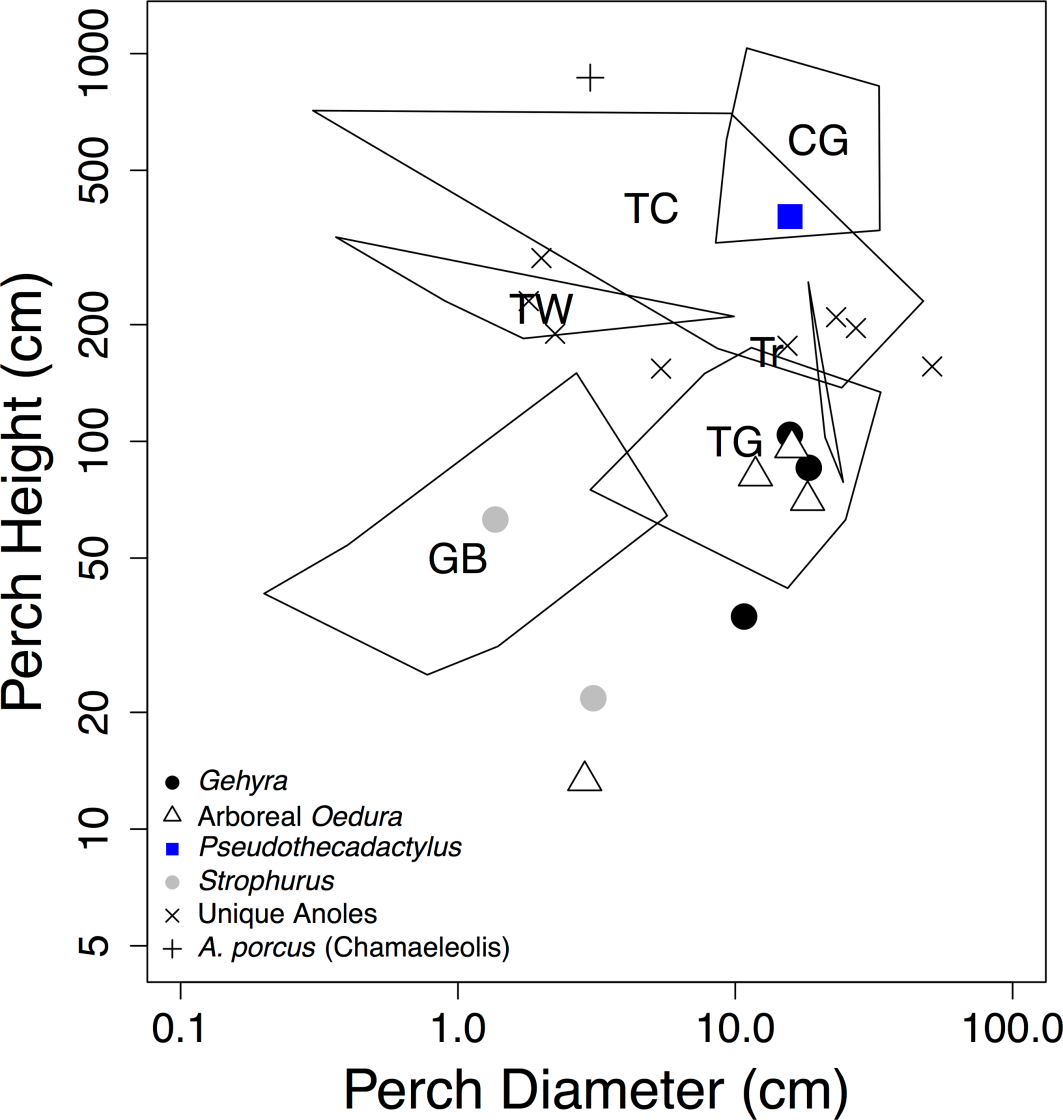
Perch heights and perch diameters of Caribbean anole ecomorphs and arboreal geckos from Queensland Australia. Polygons indicate ranges for anole ecomorphs [10, 13, 54]. Note that many geckos use perch heights and diameters that are similar to those used by anole ecomorphs. Symbols are: CG = crown giant, TC = trunk-crown, TW = twig, TG = trunk ground, GB = grass bush, *Gehyra spp*. (black circles), *Oedura spp*. and closely related *Amalosia rhombifer* (white triangles), *Pseudothecadactylus australis* (blue square), *Strophurus spp*. (grey circles), non-ecomorph (unique) anole species (X), and *Anolis porcus* from the subgenus *Chamaeleolis* (+).

**Fig 5:**
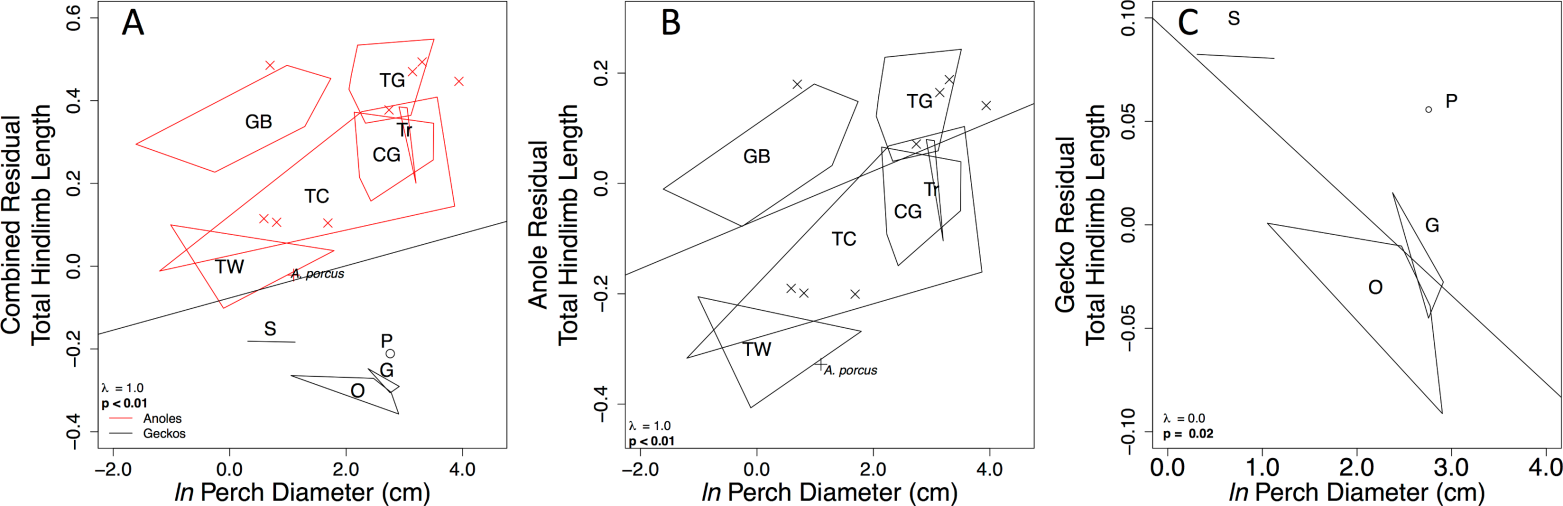
Relationships between relative limb length and perch diameter. Regression residuals of combined geckos and anoles (A), clade-specific regression residuals for anoles only (B), and geckos only (C). All plots display the PGLS correlation line, Pagel’s λ, and slope p-values. Symbols are: CG = crown giant, TC = trunk-crown, TW = twig, TG = trunk ground, GB = grass bush, non-ecomorph (unique) anole species (X), *Anolis porcus* of the subgenus *Chamaeleolis* (+), *Gehyra spp*. = G, arboreal *Oedura spp*. and closely related *Amalosia rhombifer* = O, *Pseudothecadactylus australis* = P, *Strophurus spp*. = S. Plot A illustrates a positive correlation with anole data in red and gecko data in black. Plot B illustrates the positive correlation for anoles only. Plot C displays the negative correlation for geckos only.

**Fig 6:**
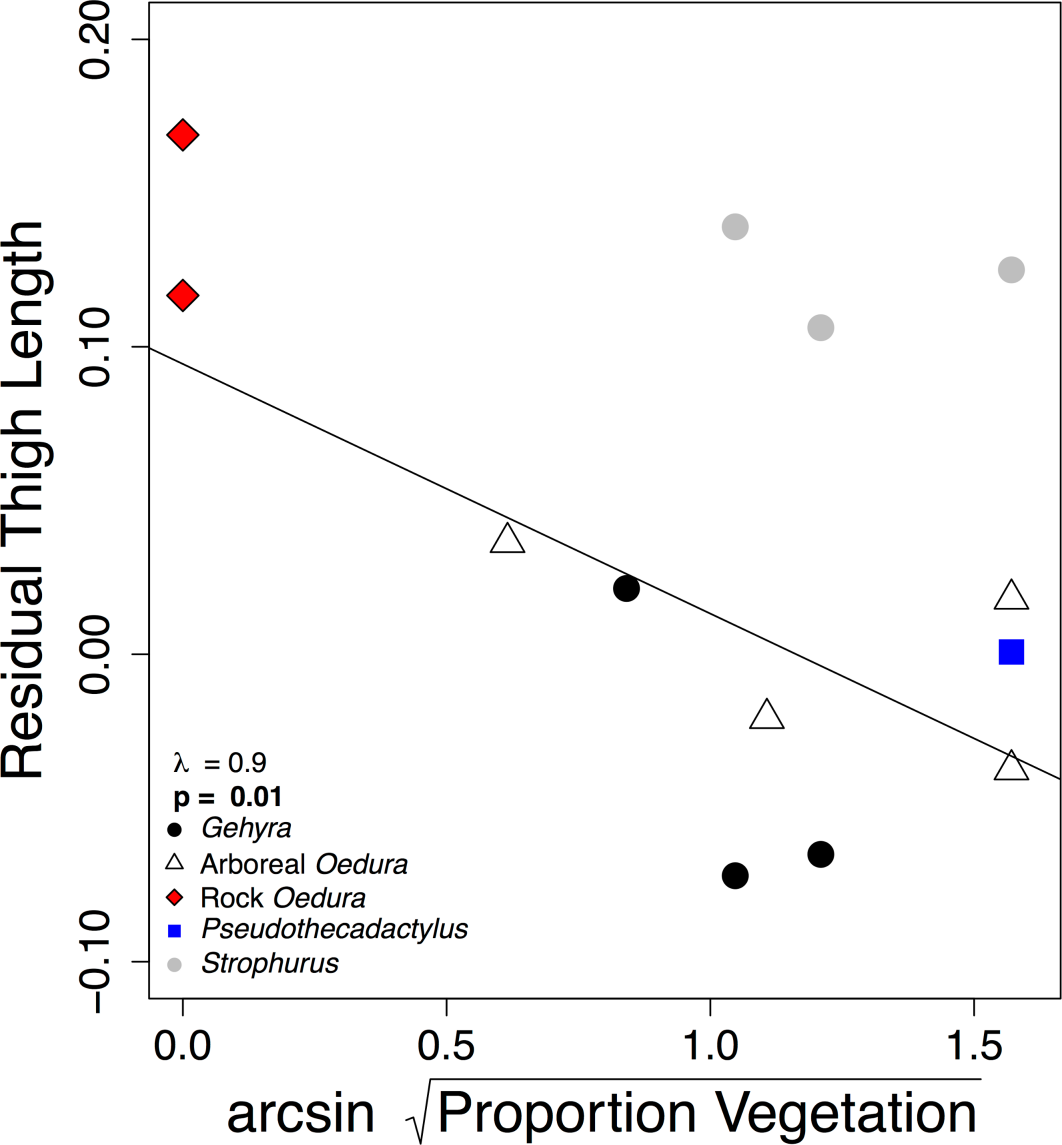
Negative correlation between residual thigh lengths and the proportion of vegetation use for each species. Brachium segment length showed a similar pattern. Symbols are: arboreal *Gehyra spp*. (black circles), arboreal *Oedura spp*. and closely related *Amalosia rhombifer* (white triangles), rock-dwelling *Oedura spp*. (red diamonds), arboreal *Pseudothecadactylus australis* (blue square), and arboreal *Strophurus spp*. (grey circles). Line = PGLS model with estimated Pagel’s λ and slope p-values.

**Fig 7:**
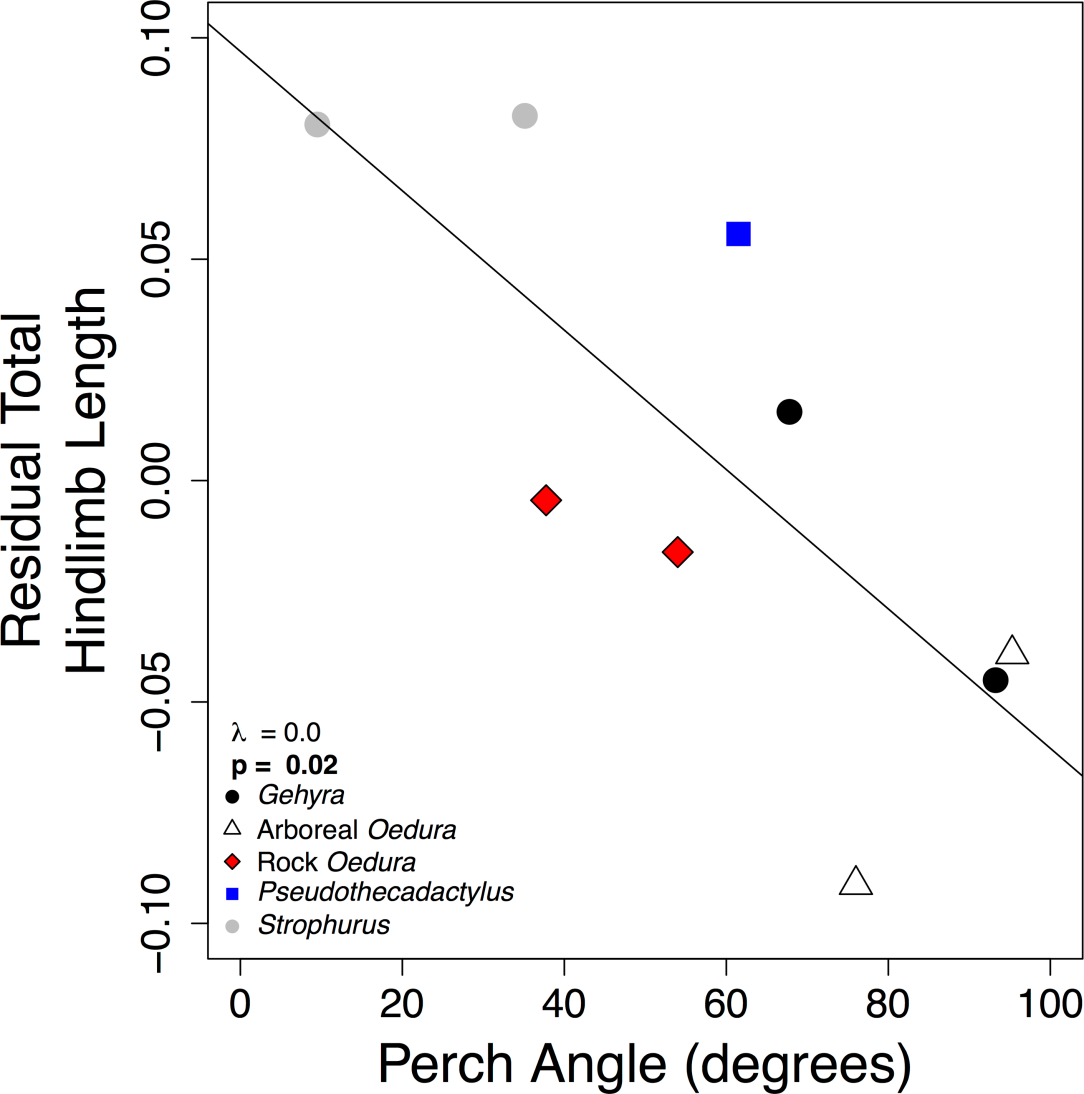
Negative correlation between perch angle and total hind limb length. Residual thigh, crus, brachium, antebrachium, and total fore segment lengths had a similar pattern. Symbols are: arboreal *Gehyra spp*. (black circles), arboreal *Oedura spp*. and closely related *Amalosia rhombifer* (white triangles), rock-dwelling *Oedura spp*. (red diamonds), arboreal *Pseudothecadactylus australis* (blue square), and arboreal *Strophurus spp*. (grey circles). Line = PGLS model with estimated Pagel’s λ and slope p-values.

For this study we used two distinct datasets, a morphological dataset and a microhabitat dataset. Our morphological dataset was comprised of 38 species of geckos and 63 species of anole (Fig 1). These data were used to compare gecko and anole limb lengths (Fig 2). We also collected a dataset of observed microhabitat patterns from 13 species of geckos from Queensland, Australia and 63 species of Caribbean anoles (Fig 3). When considering morphological and microhabitat data together, we only included species for which we had morphological and microhabitat measurements (Figs 4–7, 13 species of gecko and 63 species of anole).

### Morphology

We measured morphological characters from 38 species of pad-bearing geckos and retrieved equivalent measurements for 63 species of anole from the literature (S1 Table and [13, 54]). Gecko specimens included field caught, captive, and museum samples. Species were chosen to maximize taxonomic diversity. Using a ruler (SVL) or digital calipers (all other measurements), we externally measured snout-to-vent length (SVL); thigh length (from the point in which the hind limb enters the body to the apex of the knee); crus length (from the apex of the knee to the ankle joint); and foot length (from the center of ankle joint, measured on the dorsal side, to the tip of longest digit, toe four); brachium length (from the axilla to apex of the elbow joint), antebrachium length (from the apex of the elbow joint to the center of the wrist joint, on the dorsal side), and hand length (from the dorsal center of the wrist joint to the tip of longest digit, Fig 1). We summed our segmental fore- and hind limb lengths estimate total fore- and hind limb lengths for each individual gecko observed (S1 Table). Investigator T. Hagey collected all gecko morphological measurements. Adjustments for the various sources of our measurements, *i*.*e*. wild, captive, or museum specimens or previously published data, were not made. All of our external morphological measurements were dictated by the underlying skeletal structure and not soft tissue. We feel the potential error introduced due to variation in specimen source was likely minimal compared to the differences we observed between species.

### Microhabitat use

We examined microhabitat use for 63 species of anole and 13 species (69 individuals) of pad-bearing gecko (S1 Table). Anole information came from the literature [13, 54]. To collect gecko habitat use in the field, our field techniques were approved by the University of Idaho animal care and use committee (protocol #2012–14), the James Cook University Animal Ethics committee (JCU-A1813), and the Queensland Department of Environment and Heritage Protection (scientific collection permit #WISP11483112). Geckos were observed in Queensland, Australia during September and October 2012. Observations and collections were carried out while geckos were active, between sunset and midnight. We recorded the substratum on which animals were first sighted, categorizing them as vegetation, rock, or ground. Individuals observed on rocks were on either large boulders or rock outcrops. We calculated the proportion of observations occurring on each substrate for each species. When geckos were observed on vegetation, perch height and diameter were measured at the point of initial observation. Perch angle was recorded for all perches using a digital goniometer (Johnson model #40–6060) with measurements ranging from 0° i.e., a flat surface, 90° representing a vertical surface, and beyond 90° indicating an inverted surface. Specimens were captured by hand. After we collected morphological measurements, specimens were euthanized using MS-222 (tricaine methanesulfonate; [55]), formalin-fixed, and prepared as museum specimens. Fifty preserved specimens were submitted to the Queensland Museum (S2 Table). Individuals not euthanized were released twenty-four hours after capture at their original point of capture.

### Analyses

To conduct our analyses, we used the R Studio statistical software version 0.98.501 [56]. To ensure normality before statistical analyses, species mean perch diameter, perch height, limb lengths, and SVL were natural-log transformed. Our proportional perch-type observations were arcsine square-root transformed. Perch angle was not transformed. After calculating and natural-log transforming our species-mean limb length measurements, we extracted residuals from SVL-limb length phylogenetic generalized least squares regressions (PGLS) using the *ape* package [57], to calculate size-independent limb measurements. We used a pruned ultrametric squamate phylogeny [58]. We calculated residual limb lengths using geckos and anoles together, as well as residuals for geckos and anoles separately (see Results). To evaluate correlations between morphology and ecology, we used PGLS *via* the *caper* library [57, 59, 60] and the same phylogeny [58]. This approach also estimated Pagel’s λ, which is bounded between zero (phylogenetic relationship is not related to the residuals) and one (residuals evolve under Brownian motion).

Due to differences between our focal species and the species included in the Pyron and Burbrink phylogeny [58], we reassigned four species in the phylogeny to correspond with observed species. These changes did not greatly affect the information present in the phylogeny. *Pseudothecadactylus lindneri* became *P*. *australis*, *Afroedura karroica* became *A*. *loveridgei*, and *Geckolepis maculata* became *Afroedura hawequensis*. In the Pyron and Burbrink phylogeny *Geckolepis* is sister to *Afroedura* [58]. As a result, the only affect of substituting *Afroedura hawequensis* into *Geckolepis*, as opposed to substituting it as another species of *Afroedura*, which was not available, is that the age of the node between *A*. *hawequensis* and *A*. *loveridgei* may be overestimated. We also collected data from the recently described *Oedura bella* [61]. We assumed a similar age of divergence between *Oedura marmorata* and *O*. *bella* as Pyron and Burbrink [58] observed between *O*. *marmorata* and its sister species *O*. *gemmata*, because Oliver et al. [61, 62] hypothesized deep divergences between *O*. *marmorata* and *O*. *bella*, similar to the distance between *O*. *marmorata* and *O*. *gemmata*. Lastly, we would like to note that the Pyron and Burbrink [58] phylogeny differed from previously published phylogenies, specifically within the genus *Strophurus* [63, 64]. We retained the topology of Pyron and Burbrink [58] and suggest additional sampling to resolve conflicts.

## Results

### Morphology

Overall, geckos had relatively shorter hind limbs than anoles (Fig 2). When we calculated residual total hind limb length combining geckos and anoles, the resulting residual lengths were not normally distributed (Shapiro-Wilk normality test, **p < 0.01**, see Fig 2A inserts). All gecko species had negative residual hind limb lengths and nearly all anoles had positive residuals, resulting in a bimodal distribution (Fig 2A insert). When we calculated residual limb lengths for each group separately (Fig 2B), this approach generated normally distributed residuals for geckos (Shapiro-Wilk normality test p = 0.5, see Fig 2B inserts), yet the anole residuals still differed significantly from normal with a negative skew (Shapiro-Wilk normality test **p < 0.01**, see Fig 2B inserts).

### Microhabitat

We observed a wide variation in substratum used by geckos in Queensland. Our focal gecko species were observed using vegetation (*Amalosia rhombifer*, *Gehyra dubia*, *G*. *variegata*, *Oedura castelnaui*, *O*. *marmorata*, *Pseudothecadactylus australis*, *Strophurus ciliaris*, *S*. *krisalys*, *and S*. *williamsi*), rock (*Oedura coggeri* and *Oedura bella*), or a combination of perch types (*Gehyra robusta* and *Oedura monilis*, Fig 3).

We observed perch diameter and height values that overlapped with described anole ecomorphs (Fig 4). *Pseudothecadactylus australis* used large-diameter perches, high above the ground, very similar perch characteristics as anole trunk-crown and crown-giant ecomorphs (Fig 4). Similarly, the habitat use of most *Gehyra* and arboreal *Oedura* species overlapped with the anole trunk-ground ecomorph, as these geckos usually used vertical tree trunks (Fig 4). Geckos of the genus *Strophurus* used narrow perches near the ground, similar to grass-bush anoles (Fig 4). In addition, *Strophurus* and grass-bush anoles both also have relatively long limbs (Fig 5B and 5C). *Oedura monilis* and *Gehyra robusta* differed in microhabitat use from that of trunk-ground anoles, both using rocks and terrestrial microhabitats, in addition to arboreal perches (Figs 3 and 4). We also observed a nearly significant relationship between gecko perch height and diameter (S1 Fig).

Considering species for which we had both morphological and ecological observations, we examined the relationship between limb length and perch diameter, using residual limb lengths calculated from geckos and anoles combined and separate (Fig 5). Residual limb lengths calculated by combining anoles and geckos were positively correlated with perch diameter (Fig 5A, λ = 1.0, **p < 0.01**), suggesting that, across all focal species, species with relatively longer limbs use wider perches. Residual limb lengths calculated for each group separately suggested different patterns. Residuals of anole limb length were significantly positively correlated with perch diameter (Fig 5B, λ = 1.0, **p < 0.01**), consistent with previously published observations. Conversely, when we examined gecko limb length versus perch diameter, limb length was significantly negatively correlated with perch diameter (Fig 5C, hind total λ = 0.0, **p < 0.02**), suggesting that gecko species with relatively longer limbs use narrower perches. Closer examination revealed geckos with relatively longer thigh, crus, brachium, and antebrachium segments used narrower perches (thigh: λ = 0.0, **p < 0.01**; crus: λ = 0.0, **p < 0.01**; foot: λ = 1.0, p = 0.8; brachium: λ = 0.0, **p < 0.01**; antebrachium: λ = 0.0, **p = 0.02**; hand: λ = 1.0, p = 0.8; fore total: λ = 0.2, **p = 0.05**).

### Other Microhabitat considerations

When considering additional morphological and microhabitat relationships, including perch type and perch angle, we found that geckos we observed using vegetation had significantly shorter thigh and brachium segments and slightly longer hand segments. Hand length was weakly positively correlated with the use of vegetation (thigh: λ = 1.0, **p = 0.01**, Fig 6; crus: λ = 0.0, p = 0.4; foot: λ = 1.0, p = 0.3; hind total: λ = 0.0, p = 0.7; brachium: λ = 1.0, **p = 0.03**; antebrachium: λ = 1.0, p = 0.6; hand: λ = 1.0, p = 0.08; fore total: λ = 1.0, p = 0.4). We also observed geckos using steeper perches with relatively shorter thigh, crus, brachium, antebrachium, and total fore- and hind limb segment lengths as compared to species using more horizontal surfaces (thigh: λ = 0.0, **p = 0.01**; crus: λ = 0.0, **p = 0.04**; foot: λ = 1.0, p = 0.8; hind total: λ = 0.0, **p = 0.02**, Fig 7; brachium: λ = 0.0, **p = 0.03**; antebrachium: λ = 0.0, **p = 0.02**; hand: λ = 1.0, p = 0.8; fore total: λ = 0.4, **p = 0.03**).

## Discussion

In this study, we compared limb lengths and microhabitat use of gecko and anole lizards. Our data suggest that geckos, as a group, have relatively shorter limbs than anoles, i.e. they had a lower y-intercept, or coefficient of allometry [65]. Even after adjusting for phylogenetic non-independence in our data, when geckos and anoles were analyzed together, all limb length residuals of geckos were negative, whereas residuals of nearly all anoles were positive (Fig 2A). This overall difference in limb lengths between geckos and anoles can only be observed when analyzing these groups together (Fig 2A). However, when using residuals calculated this way in secondary analyses, such as investigations of limb length and micorhahitat use, interesting relationships maybe disguised as we discovered (Fig 5B and 5C). This a phenomenon should be considered when comparing distantly related groups.

We observed arboreal gecko species and Caribbean anoles using similar microhabitats (Fig 4). For example, *Strophurus* geckos are ecologically and morphologically similar to grass-bush anoles. Both groups use narrow perches low to the ground (Fig 4) and have relatively long limbs (Fig 5A and 5B). Further investigations comparing arboreal gecko habitat use to mainland anoles would prove very interesting. Mainland anoles, although also arboreal, to not exhibit repeated ecomorphs. We also predicted that shorter-limbed arboreal geckos would use narrower perches, similar patterns reported in anoles, *Draco*, and *Tropidurus* [6, 9–11, 14, 18]. However, we observed the opposite pattern: geckos using narrower perches had relatively longer, not shorter, limbs (Fig 5). Our results illustrate that although our focal geckos and anoles used very similar microhabitats, they have different morphological-ecological relationships. Previous studies have cited a trade-off between speed and balance to explain the negative limb length perch diameter relationship observed in other lizards [6, 16, 19, 66, 67]. Since the Queensland gecko species we observed did not display this same morphological-ecological relationship, perhaps geckos are not sensitive to the same speed and balance trade-off and negotiate narrow perches differently (see S2 Fig for additional analyses considering absolute hind limb length and perch diameter). In particular, while both geckos and anoles have adhesive pads (*Draco* and *Tropidurus* lack adhesive pads), geckos generally generate greater frictional and adhesive forces (negative normal forces) compared to anoles [26, 68, 69], possibly allowing geckos to resist better lateral forces and cling to narrow perches. Previous locomotor studies of arboreal pad-bearing lizards have found that lizards tend to lower their center of mass on narrower perches [70–74]. Future studies incorporating species limb length, adhesive capabilities, and perch diameter would be very informative.

### Other Microhabitat considerations

Studies of lizard functional morphology have also considered limb length in non-arboreal microhabitats [75–78]. For example, many studies have reported long-limbed species living on rocks, but this may not be a general trend [52, 53, 79, 80]. Similar to most previous studies, the geckos we observed more frequently on vegetation, as opposed to rocks, had significantly shorter thigh and brachium limb segments. In addition, perch diameter may not be the only variable influencing scansorial locomotion in lizards. Perch texture is likely an important factor influencing locomotion, especially of padded lizards. Although much theoretical work has been done considering surface texture and gecko performance [33, 35, 41, 42, 81, 82], few studies have examined shear forces and adhesion separately in regards to their relationship to texture and microhabitat use (but see [42]). We often observed arboreal Australian geckos using ironbark (*Eucalyptus* spp.) and paperbark (*Melaleuca* spp.) trees. Generating clinging forces on these surfaces would be difficult due to the bark of ironbark trees is very rough, with large valleys and ridges, greatly limiting the available surface area for adherence [42]. The bark of paperbark trees is smooth, but dusty and flaky, again limiting a species’ clinging ability and likely fouling their toe pads (see [83, 84]). In addition to perch texture, perch angle also likely affects scansorial lizard locomotion. Perch angle, defined the angular incline, above the horizontal, of the support, correlates with lizard adhesive toe pad size [44, 48, 85] and affects locomotor kinematics and sprint speed in some but not all lizards [25, 66, 71, 86–88]. The focal gecko species we observed using steeper perches also had relatively shorter limbs. Lizards may also navigate arboreal habitats using different locomotor strategies; for example, chameleons and twig anoles typically move along the top of single branches and twigs, whereas lacertids have been reported to “clamber” over, under, and around branches and twigs [18, 66, 89]. This clambering style may also describe how *Strophurus* and grass-bush anoles move through arboreal microhabitats. Our results suggest that there may be subtle relationships linking limb length, perch angle, and the adhesive system [6, 25, 50, 90–92]. Shorter thigh and brachium limb lengths may bring the body closer to the surface and reduce the chance of toppling off steep perches. It might be fruitful to consider microhabitats in terms of the behavior or locomotion that is associated with them, instead of categorically by composition, to improve our understanding of the biomechanics of scansorial lizards [6, 11, 66, 67, 93–98].

In this study, we measured individual limb segment lengths in addition to total limb length. This approach allowed for a more detailed understanding of the interaction between microhabitat and the locomotor system. We found differences between pad-bearing geckos and anoline lizards suggesting that although both groups have evolved similar fibrillar adhesive systems and use similar arboreal microhabitats, their relative limb lengths differ with different morphological-microhabitat relationships. These results provide an example of how morphologically and ecologically convergent systems have aspects of historical contingency and group-specific idiosyncrasies that likely impact their ecology, evolution, and adaptation.

## Supporting information

S1 Table Species means. Over the course of this study, we collected two datasets, a microhabitat dataset and a morphological data. Our anole data were compiled with the assistance of J. Losos [13, 54]. We collected gecko habitat use measurements from Queensland, Australia. 95% confidence intervals are shown in parentheses. Microhabitat column header abbreviations are PH: perch height; ArbPD: arboreal perch diameter; PercTree: proportion of observations on vegetation; PA: perch angle; N: number of individuals; Location: location of observations in Queensland; and anoline Ecomorphs: TG: trunk-ground, TC: trunk-crown, T: trunk, GB: grass-bush, TW: twig, CG: crown-giant, U: unique (non-ecomorph), CH: subgenus *Chamaeleolis*). Morphological column header abbreviations are N: number of individuals; SVL: snout-vent-length; FTotal: Total front limb length; Thigh; Crus; Foot; HTotal: Total hindlimb length; Brachium; Antebrachium; Hand; and FTotal: Total front limb length (see Fig 1).
(XLSX)

S2 Table Collected specimens deposited in the Queensland Museum. We submitted 50 wild caught lizard specimens to the Queensland Museum. Please note that species names may have been changed to follow the museum’s current species designations.
(XLSX)

S1 Fig Perch height vs perch diameter. Using a phylogenetic generalized least squares approach, we compared the relationship between perch height and perch diameter, both natural log transformed, of the perches we observed geckos using in Queensland. With an estimated λ of 0.0, we observed a near significant relationship (p = 0.07), suggesting that the high perches we observed geckos on also tended to be thick. We do not feel this weak relationship confounded our results.
(PDF)

S2 Fig Absolute limb length vs perch diameter. Using a phylogenetic generalized least squares approach, we considered the relationship between absolute hind limb length and perch diameter, both natural log transformed, for our observed Queensland geckos and Caribbean anoles. Note that both plots have the same axes. While we found no significant relationship within our focal geckos (p = 0.7), we did observe a significant relationship for anoles (p < 0.01). Overall, it appears that our observed geckos are using perches of similar diameter as compared to anoles (mostly tree trunks wider then 10 cm), but with shorter absolute limb lengths. This may suggest the limb length–perch diameter trade off observed in anoles is not present in geckos.
(PDF)
