# Supplementary figures and images for: There’s more than one way to climb a tree: Limb length and microhabitat use in lizards with toe pads
Source: PLoS One. 2017 Sep 27;12(9):e0184641. doi: 10.1371/journal.pone.0184641 (PMC5617165; doi:10.1371/journal.pone.0184641)

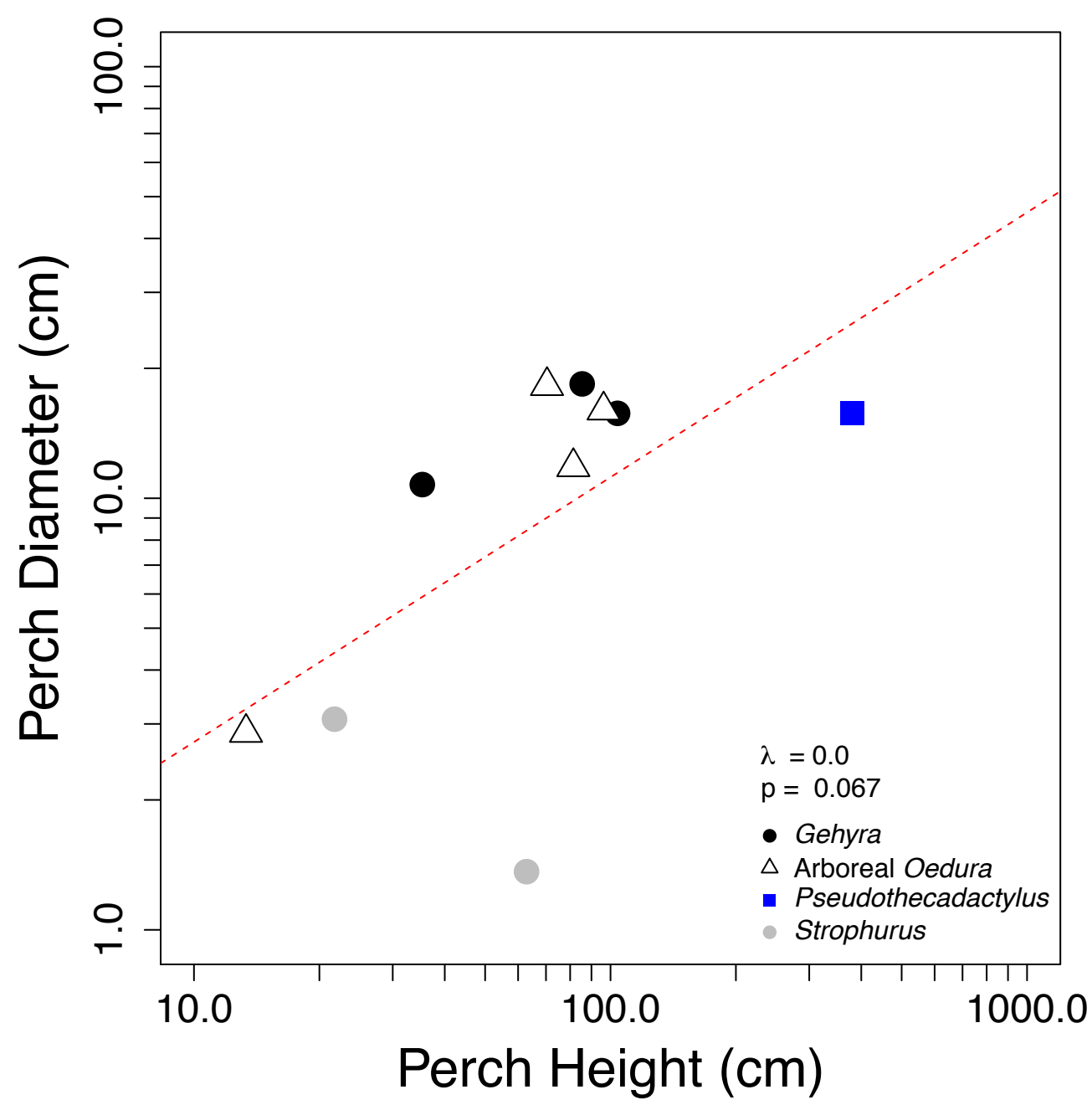

Supplement: S1 Fig — Using a phylogenetic generalized least squares approach, we compared the relationship between perch height and perch diameter, both natural log transformed, of the perches we observed geckos using in Queensland. With an estimated λ of 0.0, we observed a near significant relationship (p = 0.07), suggesting that the high perches we observed geckos on also tended to be thick. We do not feel this weak relationship confounded our results. (PDF) [file pone.0184641.s003.pdf]

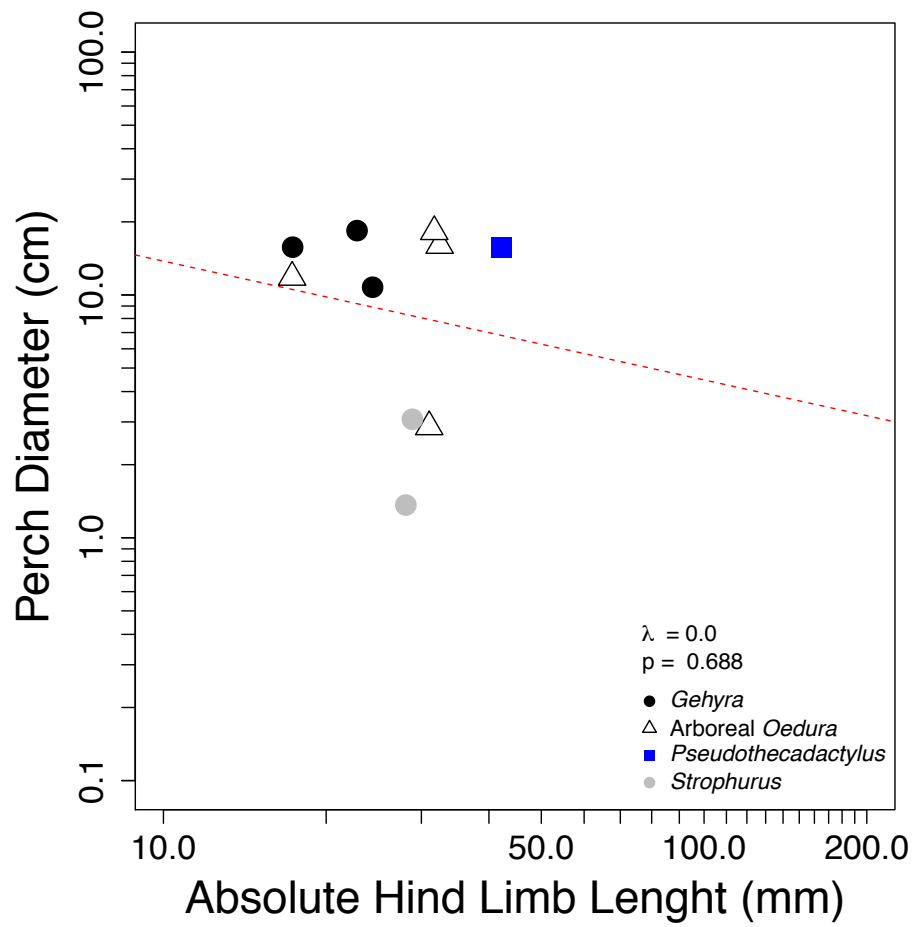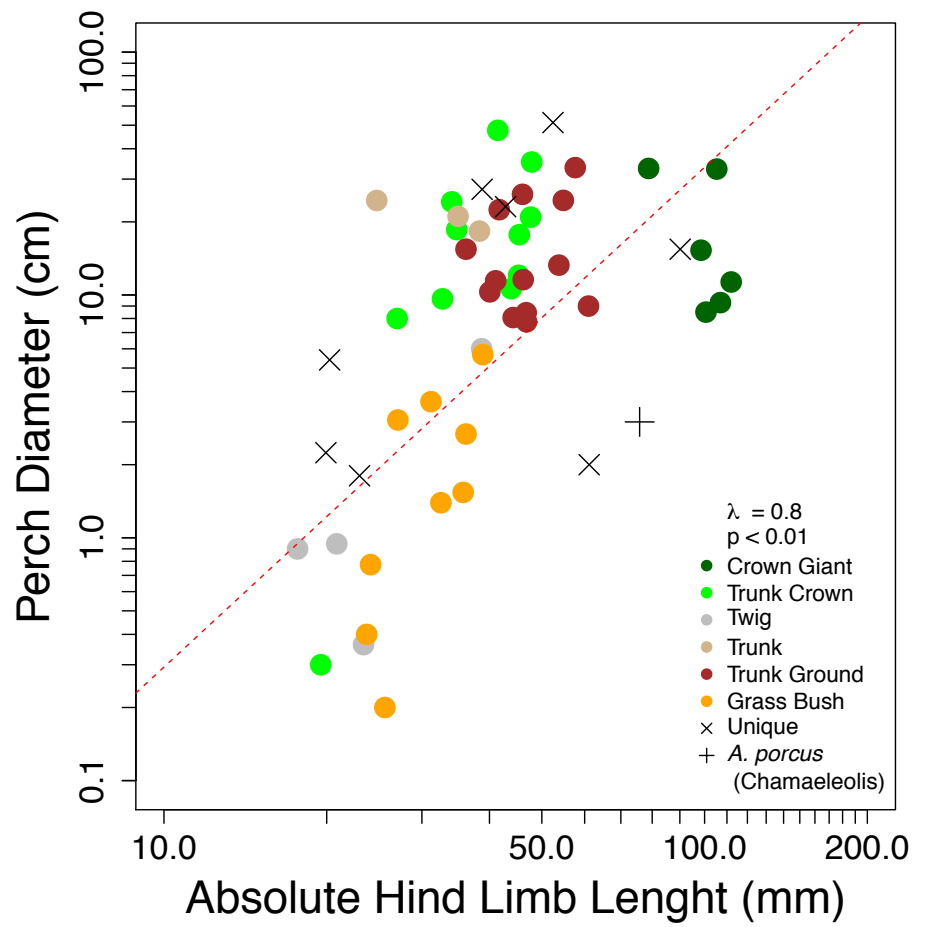

Supplement: S2 Fig — Using a phylogenetic generalized least squares approach, we considered the relationship between absolute hind limb length and perch diameter, both natural log transformed, for our observed Queensland geckos and Caribbean anoles. Note that both plots have the same axes. While we found no significant relationship within our focal geckos (p = 0.7), we did observe a significant relationship for anoles (p < 0.01). Overall, it appears that our observed geckos are using perches of similar diameter as compared to anoles (mostly tree trunks wider then 10 cm), but with shorter absolute limb lengths. This may suggest the limb length–perch diameter trade off observed in anoles is not present in geckos. (PDF) [file pone.0184641.s004.pdf]
